# Bridging the Gap: Disparities in HPV Vaccine Uptake Between In-School and Out-of-School Girls Following a Demand Generation Intervention in Ethiopia: A Cross-Sectional Study

**DOI:** 10.3390/vaccines14050405

**Published:** 2026-04-30

**Authors:** Telake Azale, Tewodros Alemayehu, Hiwot Tadesse Belay, Lisa Oot, Abebaw Gebeyehu, Zinabu Temesgen, Tinebeb Tamir, Lidya Mulat, Melkamu Ayalew, Mengistu Bogale, Liya Wondwossen

**Affiliations:** 1 Institute of Public Health, University of Gondar, Gondar P.O. Box 196, Ethiopia; atelake07@gmail.com; 2 JSI Research & Training Institute, Sunshine Tower #4, Airport Road, Kirkos Sub-City, Addis Ababa 1000, Ethiopia; tewodros.alemayehu@jsi.org (T.A.); abebaw.gebeyehu@jsi.org (A.G.); zinabu.temesgen@jsi.org (Z.T.); 3 JSI Research & Training Institute, 2733 Crystal Drive, Arlington, VA 22202, USA; lisa.oot@jsi.org; 4 Girl Effect, HGS Offices Building, 1st Floor, Bole Sub-City, Addis Ababa 1000, Ethiopia; 5 Ministry of Health—Ethiopia, 1234 Sudan Street, Addis Ababa 1000, Ethiopia; 6 Gates Foundation, Bole Sub-City, Addis Ababa 1000, Ethiopia

**Keywords:** HPV vaccination, awareness, attitude, uptake, demand intervention, out-of-school girls (OOSGs)

## Abstract

Background: Despite the availability of safe vaccines, Ethiopia’s human papillomavirus (HPV) vaccine uptake remains suboptimal, particularly among out-of-school girls (OOSGs). This study examines the effect of multi-channel demand generation messages in two districts to determine which interventions most effectively improve uptake. Methods: A convergent mixed-methods design was employed across four districts in the Somali and South Ethiopia regions, with Jigjiga and Derashe serving as intervention sites and Gode and Kolango Zuria as controls. For the quantitative component, 950 sample households were recruited using cluster sampling. The qualitative inquiry involved 27 in-depth interviews (IDIs) and 16 focus group discussions (FGDs) within the intervention sites. Results: A total of 950 caregivers and 1134 girls completed the survey. Awareness was significantly higher among caregivers (AOR: 4.42; 95% CI: (3.06, 6.39)) and girls (AOR: 7.63; 95% CI: (3.49, 16.67)) in intervention sites, as well as among in-school girls (AOR: 13.46; 95% CI: (4.09, 41.90)). The mean vaccination coverage reached 71%, with significantly higher rates in intervention sites (AOR: 4.07; 95% CI: (2.29, 7.23)) and among in-school girls (AOR: 47.16; 95% CI: (20.23, 109.9)). Interpersonal communication—via teachers, peers, community health workers and vehicle-mounted promotion—was more effective in influencing awareness, attitude and uptake. Barriers for OOSGs included limited access to vaccination sites, low campaign awareness, misconceptions and gender-related issues. Conclusions: Appropriate demand generation strategies effectively enhance HPV awareness and vaccine uptake, yet a significant equity gap remains, as only one-third of OOSGs received the vaccine compared with 85% of in-school girls. Targeted interventions are recommended for OOSGs focused on both access to service and context-specific demand creation to address this disparity.

## 1. Introduction

Cervical cancer is the second most common type of cancer in women of reproductive age, with an estimated 660,000 new cases and 350,000 deaths per year globally [1]. The high incidence in low- and middle-income countries (LMICs) is a reflection of prevailing poor access to vaccination, early detection and treatment [2,3].

Cervical cancer risk is driven by a complex interplay of socioeconomic and behavioral factors, including low educational attainment, poverty, early marriage, and limited access to healthcare [4,5]. Biologically, persistent human papilloma virus (HPV) infection is responsible for nearly 99% of all cases [6].

The global strategy to accelerate the elimination of cervical cancer as a public health problem, released in 2020, proposes an elimination threshold of four cases per 100,000 women by 2030 through triple-interventions: 90% vaccination coverage, 70% twice-lifetime cervical cancer screening coverage, and 90% treatment coverage for pre-invasive lesions and invasive cancer [7,8,9]. Prophylactic HPV vaccination provides the most effective method of primary prevention against HPV-related diseases [10].

Globally, there are effective demand interventions to increase cervical cancer awareness, knowledge, and subsequent screening or vaccination uptake [11,12,13]. Systematic reviews on the effectiveness of educational interventions that target parents and adolescents showed that they increase awareness, acceptance and uptake of the vaccine and help adolescents and parents overcome barriers [14,15,16,17]. Vaccine uptake is driven by high levels of vaccine-related literacy, access to healthcare providers as primary information sources, and overall favorable attitudes toward immunization [18]. Furthermore, strong social support from parents, peers, religious leaders, and educators—combined with high self-efficacy in overcoming logistical barriers—serves as a significant predictor of HPV vaccine acceptance [19].

Since Ethiopia introduced the HPV vaccine in 2018, approximately 6.3 million girls have been vaccinated with annual school-based campaigns as the main service delivery strategy [20]. While the program initially targeted 14-year-old girls with a two-dose regimen, the Ministry of Health transitioned to a more efficient single-dose schedule in early 2024, following evidence of its equal efficacy [21]. This was followed by a nationwide multi-age-cohort (MAC) HPV vaccination campaign in November 2024, targeting girls aged 9–14 years through schools, health facilities, and community outreaches. Despite these efforts, vaccination coverage remains a challenge. While a 2023 systematic review estimated a pooled national uptake of 42.05%, regional studies show significant variance, ranging from 15% to 66.5% [22].

This study examines the effect of multi-channel demand generation interventions implemented in two districts as part of a national HPV MAC vaccination program in Ethiopia. By evaluating the pathway from communication exposure to action, this study assesses how these interventions influence HPV vaccine awareness, attitude, and uptake among in-school and out-of-school girls aged 9–14 years in the Somali and South Ethiopia regions. Ultimately, this research aims to identify which communication strategies best improve HPV vaccine uptake to support and sustain national immunization efforts.

## 2. Methods

### 2.1. Study Design, Setting, and Period

A cross-sectional, convergent mixed-methods design was employed in the Somali and South Ethiopia regions, where the demand generation intervention was implemented. Two intervention districts, one from each region (Jigjiga in the Somali region and Derashe in South Ethiopia), and two comparison districts (Gode in the Somali region and Kolango Zuria in South Ethiopia) were studied in January 2025, two months after the HPV MAC campaign and our demand intervention was completed.

### 2.2. Population

The quantitative household survey study included randomly selected girls between 9 and 14 years and their primary caregivers residing in the four districts. Caregivers responded to questions regarding household socio-economic status, their awareness and consent, while girls reported on their own awareness, attitudes and vaccination status. The qualitative study considered both in- and out-of-school girls aged 9 to 14 years, school boys in similar age cohorts, caregivers, religious and community leaders, healthcare providers, health administrators, implementing partners, school administrators, education office heads, and community representatives residing in the two intervention districts.

### 2.3. Eligibility Criteria

Participants were recruited based on specific inclusion criteria tailored to this study’s mixed-methods design.

#### 2.3.1. Inclusion Criteria

For the quantitative component, eligibility was limited to permanent residents of the intervention and control districts, specifically adolescent girls aged 9–14 years and caregivers 18 years and older. The qualitative study involved a broader range of stakeholders within the intervention districts, including in-school and out-of-school girls (aged 9–14), school boys (aged 9–14), caregivers (18 years and older), religious and community leaders, healthcare providers, health administrators, implementing partners, and school personnel, including teachers and administrators.

#### 2.3.2. Exclusion Criteria

Individuals who had resided in the study districts for less than six months were excluded to ensure participants had sufficient exposure to demand-generation activities. Potential participants with cognitive impairment or those unable to provide informed consent were also excluded.

### 2.4. Sampling

A sample size of 924 households for the household survey was calculated based on a comparison of 2 independent proportions, assuming a 15% difference because of the intervention (60% vs. 75%), a confidence level of 95%, a power of 80%, and a design effect of 1.5.

The sample for the qualitative study was estimated based on the theoretical saturation and rules of thumb for focus group discussions and key informant interviews. The proposed sample size was 16 focus group discussions (FGDs) and 28 in-depth interviews (Is). The FGDs consisted of 6 to 9 individuals per group. Maximum variation purposive sampling was employed to include people from different backgrounds in terms of occupation, education, roles in the community, age, and experience.

### 2.5. Interventions Description

In November 2024, the Ethiopian Ministry of Health (MoH) launched a nationwide HPV MAC vaccination campaign targeting all girls aged 9–14 years. To evaluate the effect of enhanced demand generation on vaccine uptake, JSI, in partnership with Girl Effect, implemented a comprehensive, multifaceted intervention in two specific districts: Derashe district in the South Ethiopia region and Jigjiga town in the Somali region. This project supplemented the standard national advocacy and awareness-raising efforts already being conducted by the MoH and its sub-national structures across the country.

This study conducted a four-week demand intervention targeting both in-school and out-of-school girls in the two districts through several integrated channels:

School-Based Engagement: In-school girls participated in a four-week, peer-led interactive curriculum. This was supported by teachers and school ‘mini-media’ platforms to disseminate education on HPV awareness and the importance of the vaccine.

Community Outreach: Health extension workers (HEWs) and community health volunteers conducted door-to-door visits and facilitated community dialogues with caregivers and out-of-school girls. Local religious and community leaders were also engaged as key vaccine advocates.

Mass Media and Mobilization: These interpersonal efforts were reinforced by culturally resonant public service announcements on local radio and television, vehicle-mounted mobile announcements, and the strategic placement of posters and banners.

The educational content and messaging were standardized across all intervention channels, providing age-appropriate information focusing on five thematic areas:

Prevention and Vaccine Benefits: Messages provided basic information on the causal link between HPV and cervical cancer, emphasizing the vaccine’s high efficacy in preventing cancer-related morbidity and mortality.

Mitigation of Myths and Misconceptions: Interventions prioritized the active dispelling of local rumors and common misconceptions to address vaccine hesitancy and safety concerns of the girls, parents and the community members.

Targeted Demographics: Clear criteria were disseminated to define the target population, specifically focusing on the eligibility of adolescent girls aged 9–14 years to ensure the MAC parameters were understood.

Location and Scheduling: Practical information was communicated regarding the campaign’s implementation, including specific dates, times, and designated vaccination sites (both school-based and community outreach locations).

Guidance on Side Effects and Communication: Fact sheets and instructional materials were distributed to provide guidance on managing minor adverse effects and where to report if experiencing any side effects. Additionally, these materials provided adolescent girls with communication strategies to facilitate informed discussions with their parents or guardians regarding vaccine consent.

These intensified activities began four weeks prior to the MAC and continued throughout the vaccination period. During the MAC vaccination week, which was conducted for one week, the health workers from nearby health facilities provided the vaccine at schools and outreach sites and within health facilities, as planned by the MoH in all districts.

### 2.6. Data Collection Methods and Instruments

The quantitative and qualitative data were collected in parallel from 21 January 2025 to 30 January 2025. For the quantitative study, the sampling frame was obtained by listing the households with girls between 9 and 14 years. Caregivers and all eligible girls in the selected households were interviewed. We used the SurveyCTO version 2.80.3 data collection software to collect data during the household interviews. If available, vaccination cards were photographed to validate the respondents’ information.

The qualitative inquiry comprised 27 in-depth interviews (IDIs) with key informants and 16 FGDs. Key informants included local health workers, school administrators, teachers, community or religious leaders, and implementing partners directly involved in facilitating the demand-generation activities. The FGD participants were categorized into four groups to capture distinct viewpoints from both decision-makers and vaccine recipients: caregivers residing in the intervention districts, in-school adolescent girls, and out-of-school adolescent girls, identified through door-to-door mobilization and marketplace outreach. Additionally, separate discussions were conducted with schoolboys to capture the male perspectives on the vaccination program. For each qualitative inquiry, the team received consent to audio-record each session. A three-day training was implemented with data collectors and supervisors.

### 2.7. Data Processing, Analysis, and Interpretations

The quantitative data were exported from SurveyCTO to STATA version 17 for analysis. Multivariate, mixed-effects logistic regression analyses were carried out. The intervention and the non-intervention areas were compared in terms of awareness, attitude, and coverage of the HPV vaccine to examine the effect of the demand generation package and examine information on specific demand channels. Comparisons were also made between in-school and out-of-school girls and their caregivers. The qualitative data were transcribed verbatim, translated into the English language, coded, categorized, thematized, and examined for narrative insights and thematic patterns. We developed a codebook based on behavioral constructs from the Theory of Planned Behavior, the Health Belief Model, and Social Cognitive Theory about behavior and its determinants, as well as previous research on HPV vaccine uptake and the factors that enhance uptake, including knowledge, attitude, intention, beliefs, norms, and self-efficacy. Using our research objectives and questions, we designed the codebook to help answer the study’s learning questions. The Dedoose software version 9.2.22 was used for coding and analysis. The themes were identified deductively and modified in accordance with the data. As a final piece to the analysis, the team triangulated the qualitative and quantitative data to determine key learning points and the main results.

### 2.8. Ethical Considerations

Ethical approval was granted by the Ethiopian Public Health Institute (protocol number: EPHI-IRB-532-2023 on 2 December 2024), with additional permissions obtained from relevant zonal and district authorities. Participants were provided with an information sheet outlining the study’s purpose, the voluntary nature of participation, and their right to withdraw or skip questions without penalty. Written informed consent (signature or fingerprint) was obtained from all adult respondents. Furthermore, written parental or caregiver consent was obtained for each minor participant, supplemented by verbal assent from girls and boys involved in this study. To ensure confidentiality, personal identifiers were removed following qualitative participant selection, and interviews were conducted in private settings—either in participants’ homes or preferred offices—to maintain privacy.

## 3. Results

### 3.1. Socio-Demographic Characteristics of the Respondents

The quantitative interview included 950 caregivers, with 481 (50.6%) from the intervention sites. By including all eligible girls (9–14 years) in selected households, the total number of girls interviewed was 1134. Of the girls, 836 (73.7%) were in school. Most, 830 (87%), of the caregivers who participated in the quantitative study were married, and 585 (61.57%) did not have formal education (Table 1 and Table 2).

A total of 137 participants were included in the qualitative study. This consisted of 110 participants across 16 FGDs, with each group ranging from 5 to 9 individuals. Eight of the 16 FGDs (50%) were conducted specifically with adolescent girls aged 9–14. This balanced representation was designed to capture the first-hand perspective of the primary vaccine recipients regarding their awareness, attitudes, and the specific barriers they faced during the MAC campaign. In addition, 27 participants were involved in in-depth interviews as key informants (19 males and eight females). These key informants included local health workers, school administrators, teachers, religious or community leaders, and implementing partners, purposively selected due to their active roles in facilitating HPV vaccination demand generation activities; they provided insights into programmatic implementation and community-wide perceptions.

### 3.2. Knowledge of the Study Participants About the HPV Vaccine

Overall, 719 (76%) of caregivers were aware of the HPV vaccine. Caregiver awareness was significantly higher in the intervention sites at 423 (88%) compared with 296 (63%) in the non-intervention sites. Similarly, awareness among girls was 89% in the intervention sites versus 69% in the non-intervention sites.

A pronounced disparity was evident based on school status: 782 (94%) of in-school girls were aware of the vaccine, compared with only 112 (38%) of out-of-school girls. This low awareness among out-of-school girls was corroborated in the qualitative focus group discussions, where girls reported hearing about the HPV vaccine primarily through house-to-house visits by HEWs.

“No, I did not hear about it until the vaccination day when the health workers came to our home”.

An out-of-school girl FGD participant from South Ethiopia, P5.

Knowledge of where to access vaccination varied across participants. Accordingly, 424 (88%) of caregivers from the intervention sites, compared with 358 (76%) of caregivers from the non-intervention sites, were aware of the vaccination sites. This was even more pronounced among girls, where 703 (97%) of in-school girls compared with 59 (8%) of out-of-school girls knew the vaccination sites.

“Before the day the Health Extension Worker came home to vaccinate us, I had no information about where the vaccination was given. She (the HEW) gave me the vaccine near our home”.

An out-of-school girl SE: FGD_5, P1.

A multivariate logistic regression analysis indicated that caregivers from the intervention sites were 4.42 times more likely to be aware of the HPV vaccine compared with caregivers from the non-intervention sites (AOR: 4.42; 95% CI: (3.06, 6.39)) (Table 3).

A mixed-effect multilevel analysis showed that girls from the intervention sites were 7.63 times more likely to be aware of the HPV vaccine compared with girls from the non-intervention sites (AOR: 7.63; 95% CI: (3.49, 16.67)) (Table 4).

### 3.3. Sources of Information

Overall, caregivers reported that HEWs (31%), community and religious influencers (20%), and vehicle-mounted speakers (16%) were the most frequent sources of information.

Campaign awareness was driven by multiple-channel exposure, which varied based on residence and school enrollment status. Schoolgirls reported the highest frequency of message reinforcement, citing teachers and HEWs as primary sources, supplemented by community-based outreach via vehicle-mounted speakers and religious leaders.

“Yes, they (the religious leaders) teach and remind us at our church Mekane Yesus to send young girls aged 9–14 to the vaccination.”

Community representative FGD_8, P1 from SE.

The most frequent channels and sources of information about HPV differ significantly between in-school girls and out-of-school girls, reflecting their primary environments and access points. The school environment dominated information flow for in-school girls, with 744 (95.02%) of respondents citing teachers and school-based sessions as their main source of information. While in-school girls also reported receiving information from friends (111 [14.18%]) and HEWs visiting their communities (81 [10.34%]), these sources were secondary to the centralized messaging delivered within the school setting. Vehicle-mounted speakers and parents played minor roles, reaching only 4.09% and 5.36% respectively.

In contrast, out-of-school girls relied on community and peer networks. Friends acted as the leading source for 42.11% of out-of-school girls. HEWs followed closely, reaching 39.47% via home visits. Vehicle-mounted speakers also proved vital, reaching 22.81% of out-of-school girls and 41.73% of their caregivers.

Conversely, broadcasting through television and radio, and printed materials (fact sheets and posters), failed across both groups due to access and literacy barriers. These results confirm that outreach must leverage HEWs and peer networks to reach girls outside of the formal education system.

### 3.4. Participants’ Attitudes Towards HPV Vaccination

To assess the social and behavioral factors influencing HPV vaccine uptake, we examined attitudes and intention to be vaccinated, comparing intervention and non-intervention sites. In the quantitative study, caregivers and girls were asked about several key concepts, including the importance and safety of the HPV vaccine, the belief that girls should be vaccinated, the decision-making process, support from significant others, ease of access, and intention to be vaccinated (for those unvaccinated).

The majority of caregivers believed girls should be vaccinated against HPV, with a slight variation by intervention site. Accordingly, 455 (95%) of caregivers from intervention sites compared with 417 (89%) of caregivers from non-intervention sites favored vaccination of girls. The qualitative inquiry complemented this by how respondents believed they should be vaccinated.

“We learned from the health workers and teachers (community influencers) that the HPV vaccine is very important. They explained that it helps prevent a disease that can harm women and girls when they grow up. It is necessary for the girls to get vaccinated.”

A community representative woman from SE: FGD_8, P5.

“When they taught us about the health benefits of the vaccine, we supported the idea and got vaccinated. We also accept what they say to us.”

A schoolgirl FGD_1, P6 from SE.

Overall, 785 (83%) of caregivers rated the vaccine as very important. Furthermore, 422 (88%) of caregivers from the intervention sites compared with 363 (77%) of caregivers from the non-intervention sites rated the vaccine as ‘very important.’

In examining intention to be vaccinated among unvaccinated girls, 245 (75%) confirmed their intent to get the vaccine. Intention varied by site, with 101 (89%) of girls from the intervention sites stating their intention to be vaccinated, compared with 144 (67%) of girls from non-intervention sites.

Regarding vaccine safety, 76% (859) of the girls rated the vaccine as ‘moderately’ or ‘very safe.’ Perceptions of safety were notably higher in intervention sites, at 85% (486), compared with 66% (373) in non-intervention sites. A significant disparity was also observed between school-based demographics: 86% (772) of in-school girls perceived the vaccine as safe, compared with only 46% (137) of out-of-school girls. These quantitative findings were supported by qualitative data, in which most participants characterized the vaccine as beneficial and low-risk.

“It has benefits for health, but no risks at all.”

A schoolgirl FGD, P2 from the Somali region.

The majority of girls (75%) stated it was ‘moderately easy or very easy’ to get the vaccine. Among girls from intervention sites, 469 (82%) reported greater ease than girls from non-intervention sites, at 385 (69%). In-school girls also reported greater ease, at 711 (85%), than out-of-school girls, with only 143 (48%) stating it was moderately easy or very easy to get the vaccine (Table 5).

The acceptance of the vaccine was high, as reflected in the responses of the qualitative participants. The fact that religious leaders and parents supported and encouraged the girls to be vaccinated is a reflection of vaccine acceptance, according to the participants.

“The acceptance of the HPV vaccine is increasing. Those girls who were not vaccinated during the vaccination session came to inform us that they did not get the vaccine and asked us if they can be vaccinated. Even teachers and others inform us that a certain village was not vaccinated.”

KII participant community influencer, SE.

“The acceptance among teachers is at 100%; many of them even brought their daughters to be vaccinated.”

KII participant school facilitator, Jigjiga.

### 3.5. Uptake of HPV Vaccination

Overall HPV vaccine coverage in the four districts was 71% (807), with significantly higher uptake in intervention sites (81%; *n* = 462) compared with non-intervention sites (62%; 345). A similar disparity was observed between school-based demographics, where coverage among in-school girls (85%; *n* = 712) was more than double that of out-of-school girls (32%; *n* = 95). The coverage was higher among older girls, with 14-year-old girls having the highest uptake (76%) and the lowest coverage among girls 9 years of age (63%). Among the out-of-school girls (*n* = 298), about 46% (*n* = 99) of the out-of-school girls in the intervention area were vaccinated, while only 25% (*n* = 49) were vaccinated in non-intervention sites. Among the vaccinated girls (*n* = 807), school was the most frequently cited place of vaccination (90%), followed by community outreaches (8.2%), while health facilities accounted for less than 1% of the vaccinated girls during the MAC period.

Multivariate logistic regression confirmed that girls in intervention sites were four times more likely to be vaccinated than those in non-intervention areas [AOR: 4.07; 95% CI: (2.29, 7.23)]. Enrollment status was also a significant predictor, with in-school girls showing a much higher likelihood of receiving the vaccine (Table 6).

### 3.6. Barriers to Vaccination Uptake

Access to vaccination: Barriers to vaccination uptake were examined through both quantitative and qualitative methods. As part of the quantitative study, caregivers and the girls were asked about the ease of getting the vaccine. Overall, seven out of ten, 661 (70%), caregivers reported no difficulty in getting their girls vaccinated. More caregivers from the intervention sites, 417 (87%) versus 244 (52%) from non-intervention sites, reported that there were no barriers to getting their girls to the vaccination sites. This was complemented by the qualitative findings. Among the unvaccinated girls, about 35% (*n* = 116) did not receive the vaccine due to not knowing the vaccination site, while an additional 15% (*n* = 49) reported that they did not hear about the HPV vaccination campaign. This demonstrates that access to service and information is one of the major barriers to vaccination, particularly among out-of-school girls.

Access as a barrier was more consistently reported in non-intervention sites. This was particularly true for participants in Gode Woreda (non-intervention site), where about one-third of girls said it was not at all easy to get the vaccine.

Perceptions of accessibility to vaccination were high among in-school girls, their caregivers and program implementers. However, among out-of-school girls and their families, access did emerge as an important barrier. Key informant stakeholders noted that the campaign logistics favored school-based delivery, inadvertently excluding girls who were not present in the classroom. This was also conveyed by some girls who participated in the FGD.

“For those less reached groups, there are barriers to accessing the vaccine because the vaccine was mostly available in places where girls commonly gather, and these groups (unreached girls) are not present there.”

An influencer from the Somali region women’s affairs bureau, KII participant.

“Some out-of-school girls did not receive the vaccine, and I suggest that they should also be considered in future campaigns. It is important to ensure that all eligible girls, whether in school or not, have access to the vaccine.”

Community representative FGD_8, P6 from the Somali region.

Gender-related barriers: The qualitative inquiry identified gender-related barriers, including discomfort or refusal among girls to be vaccinated by a male health provider, with girls preferring to have a female vaccinator and a private space for vaccination. In addition, girls reported being teased by boys for being vaccinated, including reports that boys would refuse to marry a girl if she were vaccinated. Some fathers, serving as the main decision-makers of the families, were unwilling to let their daughters get the vaccine. This was perceived by health providers to be due to the fathers’ lack of information about the vaccine, misinformation on the vaccine’s association with infertility and promiscuity, and limited autonomy for girls to advocate for themselves and their health.

“Yes, gender barriers definitely impact HPV vaccine uptake among girls, particularly in our Muslim community. While we have female healthcare providers who administer the vaccine, there are still concerns about privacy and comfort when seeking care from male providers.”

School administrator KII participant, Somali region.

Vaccination refusal, misconceptions and hesitancy: It was also noted that some individuals refused vaccination due to various reasons, including fear of side effects (21% of unvaccinated girls), not trusting the vaccine (9%) and refusal by caregivers (4%). Stakeholders perceived the refusals to be linked to a lack of knowledge about HPV vaccination, misconceptions around the vaccine, and for girls, a fear of injection.

“Some people have expressed concerns about not accessing the vaccine, likely due to shortages. Additionally, certain communities believe that the vaccine may cause infertility, while others question why only girls are targeted. These sentiments indicate that some individuals remain suspicious of the vaccine.”

KII_ community mobilizer _from Jigjiga.

Supply-side barriers: Participants involved in the direct implementation of the campaign did note challenges related to temporary shortage of vaccines during the campaign, limited personnel for vaccination, the rushed timeline to implement activities, lengthy wait times for girls to receive the vaccine and a shortage of community mobilizers to share information before the campaign.

Other reasons: Being absent from school or home during the vaccination period was reported as a reason for not getting the vaccine. About 9% (*n* = 30) of the unvaccinated girls missed the vaccine as they were absent from school on the vaccination day. The qualitative inquiry revealed that most of the out-of-school girls spend the working days at their parents’ farms, either keeping birds away from birds or doing harvest work. An out-of-school girl’s reason for not having heard about the HPV vaccine illustrates the lack of access to information that rural girls face.

“I was not at home. I was on the farm and the farm is very far from our house.”

An out-of-school girl: FGD_6, P2 from South Ethiopia, Yayibe kebele.

## 4. Discussion

This convergent mixed-methods evaluation study assessed the effectiveness of a demand generation intervention in improving HPV vaccine uptake by raising awareness, influencing attitudes, and dispelling misconceptions. The intervention employed multiple strategies—tailoring messages, channels, and media—to address diverse population segments based on residence, education levels, and overall access to services.

The primary finding is that the demand generation interventions significantly increased awareness, brought favorable attitudes and led to higher HPV vaccine uptake in the intervention sites.

Quantitatively, significant differences were observed across the groups for both awareness (caregivers and girls) and vaccine uptake (girls). Specifically, the demand generation activities accounted for a greater than 20% difference in awareness. Although impossible to fully discern, the school-based education program was very successful in improving uptake of the HPV vaccine among in-school girls. Evidence shows that there is a very high correlation between vaccine uptake and knowledge, attitude, and acceptance of an intervention [23,24]. The role of educational intervention in increasing knowledge on HPV in this study has been supported by several studies included in a systematic review [25].

However, despite seeing significant improvements, the findings clearly demonstrate that out-of-school girls and their caregivers were not reached to the same extent as their in-school peers, and this resulted in lower uptake among the out-of-school girls. Two out of three out-of-school girls (68%) were not vaccinated, while only 15% of the in-school girls were unvaccinated, showing unequal utilization of the vaccine. This wide knowledge and vaccine uptake gap is likely due to the out-of-school girls’ having limited awareness about the vaccine, not knowing where to get it, misconceptions about the vaccine, extensive engagement in household chores, and less family support (as caregivers are also less aware of the vaccine). Similar findings were found in other previous studies in Ethiopia [26,27].

The service delivery approach, being mainly school-based, also contributed to missing a majority of the out-of-school girls, as the vaccination sites were inconvenient to these girls. Provision of HPV vaccination routinely, either integrated with routine immunization or periodic outreach focused on HPV vaccination, has been found to be effective in other settings. Identifying and vaccinating out-of-school girls needs meticulous planning, including enumerating and tracing where the girls are living, tailored demand generation, and ensuring the vaccination sites are accessible through organizing regular outreaches and availing routine/daily availability of the vaccine within health facilities [28,29]. Mixed delivery approach of vaccination at schools and health facilities is reported to be the most successful [30,31].

Some of the challenges were transient and related to the health system, such as the vaccines having a short shelf-life and an insufficient number of service providers. Such challenges are common in low- and middle-income countries [32]. The discrepancy between vaccine awareness and actual uptake among out-of-school girls highlights a critical gap in accessibility, suggesting that positive perception alone is insufficient to overcome logistical or physical barriers to vaccination [30,31].

This study implies that vaccine delivery strategies to address out-of-school girls include health facilities, community outreach approaches, and religious institutions. The annual, mainly school-based vaccine delivery strategies are not sufficient to successfully identify and vaccinate out-of-school girls.

## 5. Conclusions and Recommendations

Overall, the demand generation interventions did work to increase awareness and attitudes and improve the coverage of the HPV vaccine, as significant differences were observed between the intervention and non-intervention sites in all the outcomes. This study revealed a stark disparity in vaccine uptake: 85% coverage was achieved among in-school girls, compared with just 33% among those not enrolled in school. This highlights a critical gap in reaching the out-of-school population within the 9–14 age cohort. The importance of employing multiple message transmission strategies in addressing different segments of the population has been underscored. Communication channels involving inter-personal communication (such as teachers and peers for in-school girls and house visits by community health workers/HEWs plus vehicle-mounted community mobilization for out-of-school girls) were found to be the most effective channels for reaching girls and their families with information about HPV vaccination. Messages passed through mass media (such as radio and television) had limited reach and effect on the girls directly. This study underscores the importance of interpersonal communication as the most effective means of demand generation; however, as IPC approaches can be more expensive and resource-intensive, strategic planning to tailor IPC interventions based on localized need will be important.

Our findings underscore the necessity of a school-based vaccination framework integrated with targeted outreach for out-of-school girls. While school-based demand generation involving teachers should continue to be the primary strategy for reaching the majority of girls with the HPV vaccine, reaching non-enrolled populations requires extended lead times and close alignment with interventions designed to enhance awareness and simplify access. To reach out-of-school girls, tailored messages targeting girls and their parents through house visits and community dialogues/discussions should be part of future vaccination plans. Furthermore, our study demonstrates a critical need to address gender-related barriers hindering HPV vaccine uptake. To mitigate these challenges, demand-side interventions must expand beyond adolescent girls to engage boys, fathers, and other influential community decision-makers. In conclusion, to bridge the gap in HPV vaccination coverage between in-school and out-of-school girls, there is a need to provide HPV vaccination as part of routine service delivery, through static services at health facilities as well as through outreach services. Ideally, these services are integrated with adolescent health or routine immunization services and linked with tailored demand creation.

## Figures and Tables

**Table 1:** Demographic characteristics of the caregivers of the study districts (*n* = 950).

Variable		Derashe (*n* = 239)	Kolango (*n* = 233)	Jigjiga (*n* = 242)	Gode (*n* = 236)	Total (*n* = 950)
Place of residence	Rural	100%	100%	1%	0%	50%
	Urban	0%	0%	99%	100%	50%
Marital status	Married/living together	92%	91%	74%	93%	87%
	Single	2%	3%	5%	4%	3%
	Divorced/separated	2%	1%	17%	2%	5%
	Widowed	3%	5%	5%	2%	4%
Religion	Orthodox	39%	39%	12%	0%	23%
	Muslim	0%	0%	88%	100%	47%
	Protestant	61%	61%	0%	0%	30%
Caregivers’ education	No education	75%	55%	41%	75%	62%
	Elementary (1–8)/read and write	20%	30%	37%	17%	26%
	Secondary (9–12)	3%	6%	17%	5%	8%
	Tech/voc level	2%	5%	2%	1%	2%
	Higher level	0%	3%	4%	3%	2%
Caregivers’ occupation	Unemployed	0%	0%	2%	7%	2%
	Housewife	69%	52%	64%	68%	63%
	Farmer	28%	26%	0%	0%	13%
	Business person	1%	9%	12%	14%	9%
	Civil servant	0%	6%	8%	2%	4%
	Teacher	1%	3%	1%	2%	2%
	Private worker	0%	0%	3%	2%	1%
	Daily laborer	0%	2%	5%	4%	3%
	Student	1%	1%	2%	0%	1%
	Other	0%	0%	2%	1%	1%
Wealth index	Low	58%	76%	1%	9%	36%
	Middle	26%	13%	14%	72%	31%
	High	16%	11%	85%	19%	33%

**Table 2:** Demographic characteristics of the girls in the study districts (*n* = 950).

Variables	Category	Derashe (*n* = 279)	Kolango (*n* = 285)	Jigjiga (*n* = 294)	Gode (*n* = 276)	Total (*n* = 1134)
Age (years)	9	13%	14%	17%	12%	14%
	10	19%	17%	15%	10%	15%
	11	13%	19%	10%	15%	14%
	12	20%	20%	17%	22%	20%
	13	16%	16%	20%	15%	17%
	14	19%	14%	20%	25%	20%
School status	Out of school	25%	45%	10%	26%	26%
	In school	75%	55%	90%	74%	74%
Grade level	Never been to school	0.0%	0.0%	0.4%	0.5%	0.2%
	Elementary (1–4)	54%	70%	48%	50%	54%
	Elementary (5–8)	46%	30%	51%	46%	45%
	Secondary (9–12)	0.0%	0.0%	0.8%	3.7%	1.2%

**Table 3:** Multivariate logistic regression analysis for factors associated with awareness of caregivers about HPV vaccine (*n* = 950).

Variable	Awareness of Caregiver	Odds Ratio	[95% CI]	
Program area	Control	1	1	1
	Intervention	**4.42**	3.06	6.39 ***
Residence	Rural	1	1	1
	Urban	1.45	0.41	5.16
Education of caregiver	No education	1	1	1
	Elementary (1–8)/read and write	0.82	0.56	1.20
	Secondary and above	1.16	0.64	2.08
Religion of caregiver	Orthodox	1	1	1
	Muslim	1.51	0.43	5.22
	Protestant	1.28	0.83	1.98
Wealth index	Low	1	1	1
	Middle	**2.27**	1.40	3.66 ***
	High	1.97	1.14	3.42

*** Variables shown in bold indicate statistical significance at the *p* < 0.05 level in the multivariable model.

**Table 4:** Mixed-effect multivariate logistic regression analysis showing the association between awareness of girls and demographic characteristics.

Variable	Awareness of Girls	AOR	[95% CI]	
Program area	Control	1	1	1
	Intervention site	**7.63**	3.49	16.67 ***
Out/in school	Out of school	1	1	1
	In school	**134.60**	44.09	410.90 ***
Residence	Rural	1	1	1
	Urban	30.21	0.17	5230.41
Caregiver education	No education	1	1	1
	Elementary (1–8)/read and write	0.66	0.33	1.34
	Secondary and above	1.99	0.70	5.66
Caregiver religion	Orthodox	1	1	1
	Muslim	0.05	0.00	9.91
	Protestant	0.76	0.33	1.72
Wealth index	Low	1	1	1
	Middle	1.66	0.64	4.27
	High	0.32	0.11	0.90

*** Variables shown in bold indicate statistical significance at the *p* < 0.05 level in the multivariable model.

**Table 5:** Attitudes and perceptions of girls towards the HPV vaccine (*n* = 1134).

Variable	Control (*n* = 561)	Intervention (*n* = 573)	Out-of-School Girls (*n* = 298)	In-School Girls (*n* = 836)	Total (*n* = 1134)
Level of importance					
Not at all important	4.28%	0.17%	6.38%	0.72%	2.20%
A little important	8.56%	6.98%	18.12%	4.07%	7.76%
Moderately important & very important	76.30%	89.53%	63.42%	89.95%	83.0%
I am not sure (Don’t know)	10.87%	3.32%	12.08%	5.26%	7.05%
Safety of vaccine					
Not at all safe	4.99%	0.35%	7.38%	0.96%	2.65%
A little safe	10.7%	10.12%	24.50%	5.38%	10.41%
Moderately safe & very safe	66.50%	84.82%	45.97%	86.36%	75.75%
Don’t know	17.83%	4.71%	22.15%	7.30%	11.20%
Support from family					
No	22.28%	4.20%	27.85%	8.73%	13.76%
Yes	77.72%	94.59%	72.15%	91.27%	86.24%
Support from friends					
No	28.70%	8.60%	34.23%	13.00%	18.20%
Yes	71.30%	91.45%	65.77%	87.08%	81.48%
Ease of access					
Not at all easy	8.02%	2.27%	14.77%	1.67%	5.11%
A little easy	23.35%	15.88%	37.25%	13.28%	19.58%
Moderately easy & very easy	68.63%	81.85%	48.00%	85.05%	75.31%

**Table 6:** Multivariate logistic regression analysis showing factors associated with HPV vaccine uptake among girls (*n* = 807).

Variable	Vaccinated Girls	AOR	[95% CI]	
Program area	Control site	1	1	1
	Intervention site	**4.07**	2.29	7.23
Girl schooling	Out of school	1	1	1
	In school	**47.16**	20.23	109.9
Residence	Rural	1	1	1
	Urban	0.23	0.06	0.94
Caregiver education	No education	1	1	1
	Elementary (1–8)/read and write	0.62	0.35	1.08
	Secondary and above	0.50	0.24	1.05
Caregiver religion	Orthodox	1	1	1
	Muslim	3.50	0.99	12.38
	Protestant	0.59	0.29	1.16
Wealth index	Low	1		1
	Middle	1.34	0.64	2.80
	High	0.48	0.21	1.08

Variables shown in bold indicate statistical significance at the *p* < 0.05 level in the multivariable model.

## Data Availability

The data presented in this study are available upon request from the corresponding author due to the sensitive nature of the information collected from adolescent participants and Institutional Review Board (IRB) confidentiality requirements.
